# Mini-max feedback control as a computational theory of sensorimotor control in the presence of structural uncertainty

**DOI:** 10.3389/fncom.2014.00119

**Published:** 2014-09-24

**Authors:** Yuki Ueyama

**Affiliations:** Department of Rehabilitation Engineering, Research Institute of National Rehabilitation Center for Persons with DisabilitiesTokorozawa, Japan

**Keywords:** reaching, motor control, H^∞^ control, robust control, optimal feedback control, feedback gain, stiffness tuning, force field

## Abstract

We propose a mini-max feedback control (MMFC) model as a robust approach to human motor control under conditions of uncertain dynamics, such as structural uncertainty. The MMFC model is an expansion of the optimal feedback control (OFC) model. According to this scheme, motor commands are generated to minimize the maximal cost, based on an assumption of worst-case uncertainty, characterized by familiarity with novel dynamics. We simulated linear dynamic systems with different types of force fields–stable and unstable dynamics–and compared the performance of MMFC to that of OFC. MMFC delivered better performance than OFC in terms of stability and the achievement of tasks. Moreover, the gain in positional feedback with the MMFC model in the unstable dynamics was tuned to the direction of instability. It is assumed that the shape modulations of the gain in positional feedback in unstable dynamics played the same role as that played by end-point stiffness observed in human studies. Accordingly, we suggest that MMFC is a plausible model that predicts motor behavior under conditions of uncertain dynamics.

## Introduction

**Figure 1:**
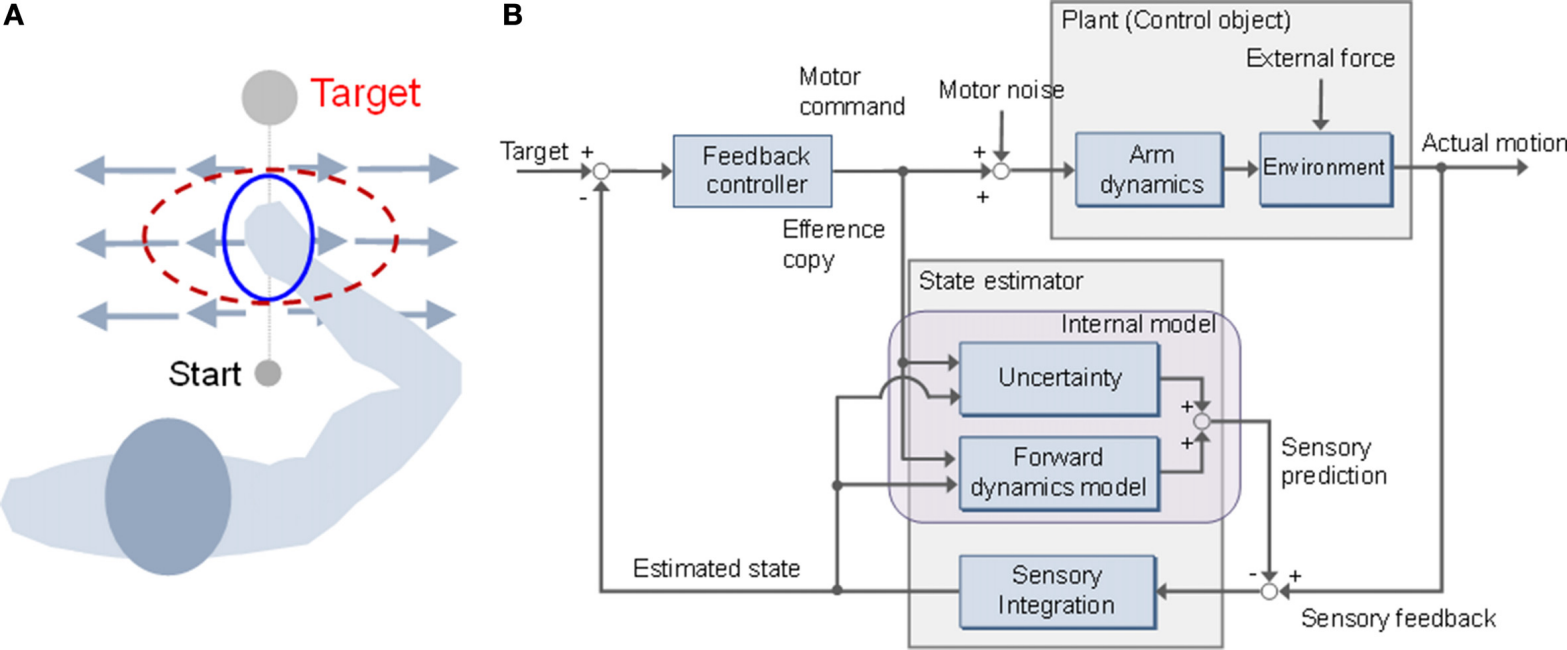
**Stiffness modulation and mini-max feedback control (MMFC)**. **(A)** Adaptation of stiffness geometry to unstable dynamics. The stiffness changes to the red dotted ellipse from the initial blue solid form. The long and short axes of the ellipse represent the directions of maximal and minimal stiffness, respectively. **(B)** Block diagram of MMFC with uncertainty.

It is necessary to interact with various environments to learn how to use tools and to participate in unfamiliar sports, such as tennis and swimming. Skilled actions are achieved via interactive forces, based on human compensation. A considerable amount of research has focused on arm movement, to investigate learning mechanisms for adaptation to perturbed limb dynamics. It has been suggested that there are different mechanisms for adapting to stable and unstable dynamics (Franklin et al., 2003a,b; Osu et al., 2003). Under conditions where the dynamics are stable, it is possible to learn the forces necessary to compensate for perturbed dynamics in a feed-forward manner (Shadmehr and Mussa-Ivaldi, 1994). However, unstable dynamics make it necessary to learn the optimal mechanical impedance as the magnitude, shape, and orientation of the end-point stiffness (Figure 1A) (Burdet et al., 2001). Although an internal model can compensate for both stable and unstable dynamics, mechanisms have been identified for adapting to different approaches (Franklin et al., 2003b; Osu et al., 2003). Osu et al. reported that an inverse dynamics model that controlled the net joint torque performed well in a stable environment. However, in an unstable environment, the inverse dynamics model functions in parallel with an impedance controller to compensate for a consistent perturbing force (Osu et al., 2003). It has also been suggested that the impedance controller assists in the formation of the inverse dynamics model and contributes to improved stability (Franklin et al., 2003b). Both approaches are used selectively and combined in accordance with environmental dynamics.

Optimal feedback control (OFC) theory (Todorov and Jordan, 2002), which has been supported by the results of experimental and simulation studies (Liu and Todorov, 2007; Lockhart and Ting, 2007; Izawa and Shadmehr, 2008; Izawa et al., 2008; Nagengast et al., 2009; Pruszynski et al., 2011; Ueyama and Miyashita, 2013, 2014), suggests that the central nervous system sets up feedback controllers that continuously convert sensory input into motor output, optimally tuned to the task at hand, by trading off energy consumption with constraints, such as accuracy, on performance. According to OFC, trajectory planning is not required because the problems of motor planning and control are combined. An important feature of the model is the concept of minimum intervention: i.e., setting up feedback controllers only to correct variation deleterious to the task (Wolpert and Flanagan, 2010). For example, in a tennis serve, variation in the azimuth angle of the racket head should be corrected far less strongly than variation in the elevation angle, because the azimuthal variation has little effect on whether the ball will land in the court, whereas elevation variability can threaten the goal of landing the ball in the court. OFC is based on a linear-quadratic-Gaussian (LQG) design, which is used to describe uncertain linear systems disturbed by additive white Gaussian noise with imperfect state information (Todorov, 2005). However, a precise forward dynamics model is required. The control and sensory noise must be modeled as Gaussian statistics; however, real-world sensorimotor uncertainties are represented by non-Gaussian distributions (Orban and Wolpert, 2011). In the engineering field, robust control design has been used in various situations, because it does not require precise dynamic models for the control objects. It is necessary to represent the uncertainties of the dynamics model in a quantitatively expressible form, because the objective of robust control is to configure a control system to allow for such uncertainties. There are essentially two ways of representing the uncertainties: as unstructural or structural uncertainties. An unstructural uncertainty is represented as a perturbation of the transform function in the frequency domain. In contrast, a structural uncertainty is represented by an additive disturbance combined with the process and sensory noise, such as environmental dynamics, in the state-space model. H^∞^ control is a robust control technique that addresses the issue of worst-case controller design for linear plants subjected to unknown additive disturbances and plant uncertainties, including problems of disturbance attenuation and model matching and tracking (Djouadi and Zames, 2002). Furthermore, the role of game theory in the design of robust controllers, such as H^∞^ control, has also been recognized (Anderson and Moore, 1979; Bernhard, 1995), with the terminology “mini-max controller” adapted from statistical decision theory (Savage, 1955). Moreover, the brain might also be treated as an integrated robust control system in which components for sensing, computation, and decision are useful primarily to the extent that they affect action (Doyle and Csete, 2011).

Here, we applied a mini-max feedback controller (MMFC) to a sensorimotor control problem with environmental dynamics as a structural uncertainty (Figure 1B). MMFC operates as an extended model of OFC, by incorporating prior influence characterized by familiarity with novel dynamics. Such expansion of motor control and planning models has been recognized as a major factor in movement generalization (Yan et al., 2013). We performed numerical simulations and compared the performance of MMFC with that of OFC, as a reference, in different types of force fields with stable and unstable dynamics. In our simulations, mini-max feedback control showed better performance than OFC under conditions of dynamics, and could predict the impedance modulation in unstable dynamics to improve stability. These observations suggest that MMFC is a plausible model that predicts behaviors under structural uncertainty. Preliminary results of this study were presented in the proceedings of a conference (Ueyama and Miyashita, 2011).

## Materials and methods

In this paper, the solution to a robust control problem was obtained via a mini-max approach applied to dynamic game problems (Başar and Bernhard, 1995). We modeled the dynamics as structural uncertainties to apply the mini-max approach; the simulations used simple Euler integration with a 5 ms sampling time.

### Mini-max control problem

The MMFC problem requires a control object to be represented as a generalized plant model. We provide the model with structural uncertainty, and the solution is obtained by minimizing energy consumption under conditions of maximal uncertainty.

#### Problem definition

The dynamics of a system are described by the following equation:

(1) $$\{\begin{array}{l} x_{k\;+\;1}=Ax_{k}+Bu_{k}+D{\bar{w}}_{k}\\ \;y_{k}=Cx_{k}+{\bar{v}}_{k} \end{array},$$

where **x**_*k*_, **u**_*k*_, and **y**_*k*_ are the state, input, and output vectors, respectively, at time step *k* (*k* = 0, 1, …, *N* − 1), and the dynamics are described by the three matrices, **A**, **B**, and **C**. **w**_*k*_ denotes a disturbance vector, and **v**_*k*_ is a sensory noise vector, represented as zero-mean Gaussian white noise with unity covariance. The system can be rewritten as follows when a disturbance, such as an environmental perturbation or motor noise, affects the dynamics:

(2) $$\{\begin{array}{l} x_{k\;+\;1}=\left(A+\Delta_{A}\right)x_{k}+\left(B+\Delta_{B}\right)u_{k}\\ \;y_{k}=\left(C+\Delta_{c}\right)x_{k}+{\bar{v}}_{k} \end{array},$$

where Δ_**A**_, Δ_**B**_, and Δ_**C**_ represent disturbances corresponding to the state, input, and output, respectively. A disturbance should be modeled as an uncertainty in the internal model. Thus, these disturbances are assumed to have the following form:

$$\Delta_{A}=D_{a}F_{ak}E_{a},\Delta_{B}=D_{b}F_{bk}E_{b},\Delta_{C}=D_{c}F_{ck}E_{c},$$

where **D**_*a*_, **D**_*b*_, **D**_*c*_, **E**_*a*_, **E**_*b*_, and **E**_*c*_ are constant matrices, and **F**_*ak*_, **F**_*bk*_, and **F**_*ck*_ are time-varying matrices satisfying the following conditions: **F**^*T*^_*ak*_ **F**_*ak*_ < **I**, **F**^*T*^_*bk*_ **F**_*bk*_ < **I**, **F**^*T*^_*ck*_ **F**_*ck*_ < **I**. The system can be transformed to the equivalent system as follows:

$$\{\begin{array}{l} x_{k\;+\;1}=Ax_{k}+Bu_{k}+\left[\begin{array}{lll} D_{a} & D_{b} & 0 \end{array}\right]\;{\bar{w}}_{k}\\ \;y_{k}=Cx_{k}+\left[\begin{array}{lll} 0 & 0 & D_{c} \end{array}\right]{\bar{\;w}}_{k}+D_{y}v_{k} \end{array},$$

where,

$${\bar{w}}_{k}=\left[\begin{array}{ccc} F_{ak} & 0 & 0\\ 0 & F_{bk} & 0\\ 0 & 0 & F_{ck} \end{array}\right]\;z_{k},\;z_{k}=\left[\begin{array}{c} E_{a}\\ 0\\ E_{c} \end{array}\right]\;x_{k}+\;\left[\begin{array}{c} 0\\ E_{b}\\ 0 \end{array}\right]\;u_{k}.$$

Here, **z**_*k*_ denotes the regulated output vector. Then, a system with structural uncertainty can be reduced to the following form, known as a generalized plant model (Zhou and Doyle, 1998):

(3) $$\{\begin{array}{c} x_{k+1}=Ax_{k}+Bu_{k}+Dw_{k}\\ \;z_{k}=Hx_{k}+Gu_{k}\;\\ \;y_{k}=Cx_{k}\;+Ew_{k} \end{array},$$

where,

$$\begin{array}{l} H=\left[\begin{array}{c} E_{a}\\ 0\\ E_{c} \end{array}\right],\;G=\left[\begin{array}{c} 0\\ E_{b}\\ 0 \end{array}\right],D=\left[\begin{array}{cccc} D_{a} & D_{b} & 0 & 0 \end{array}\right],\\ E=\left[\begin{array}{cccc} 0 & 0 & D_{c} & D_{y} \end{array}\right],\;w_{k}=\left[\begin{array}{c} {\bar{w}}_{k}\\ v_{k} \end{array}\right]. \end{array}$$

According to OFC, the cost function *J*(**u**) is given by,

(4) $$J(u)=\sum_{k\;=\;1}^{N\;-\;1}z_{k}^{T}z_{k}+x_{N}^{T}Q_{N}x_{N},$$

where **Q**_*N*_ denotes a terminal state cost weight matrix. Instead, our proposed model adopts the cost function *J*_γ_(**u**,**w**), given by the following equation:

(5) $$J_{\gamma }(u,w)=J(u)-\gamma^{2}\sum_{k\;=\;1}^{N\;-\;1}w_{k}^{T}w_{k},$$

where γ is a scalar parameter representing the level of disturbance attenuation. The objective of robust control is to determine the appropriate input for a worst-case disturbance. Thus, the robust control problem is related to the mini-max problem of minimizing the input **u** for a maximized disturbance **w**:

$$\underset{\text{u}}{\inf}\underset{\text{w}}{\;\sup}J_{\gamma }(u,w).$$

This cost function requires a task to be achieved with minimal energy consumption for the worst case of uncertainty as the maximized disturbance, in a manner analogous to the OFC problem: LQG design, which is described by a quadratic cost, and gives the solution as a combination of the feedback control law and a state estimator.

#### Solution

As in the LQG design, a solution of the MMFC problem can be written in a state feedback form:

(6) $$u_{k}=-L_{k}{\hat{x}}_{k},$$

where $\hat{x}$_*k*_ and **L***_*k*_* are the estimated state and feedback gain, respectively. The estimated state and feedback gain are computed from two discrete Riccati differential equations of the following form:

$$\begin{array}{l} \;M_{k}=Q_{k}+A^{T}{\left(M_{k\;+\;1}^{-1}+BB^{T}-\gamma^{-2}DD^{T}\right)}^{-1}A\\ \text{with }M_{N}=Q_{N},\\ \;\Sigma_{k\;+\;1}=A{\left(\Sigma_{k}^{-1}+C^{T}N^{-1}C-\gamma^{-2}Q_{k}\right)}^{-1}A^{T}+DD^{T}\\ \text{with }\Sigma_{1}=Q_{0}^{-1}, \end{array}$$

where **M**_*k*_ and **Σ**_*k*_ denote the solutions of the Riccati equations obtained by the respective backward and forward time calculations. Here, we adopt the following assumptions to simplify the derivations:

$$G^{T}G=I,H^{T}G=0,EE^{T}=N,H^{T}H=Q_{k}.$$

These assumptions do not affect the generalizability, and they allow describing equations in simple forms, maintaining consistency with the OFC. Using these solutions, the feedback gain and estimated state are given by the following:

(7) $$\begin{array}{l} L_{k}=B^{T}{\left(M_{k\;+\;1}^{-1}+BB^{T}-\gamma^{-2}DD^{T}\right)}^{-1}\\ \;A{\left(I-\gamma^{-2}\Sigma_{k}M_{k}\right)}^{-1}, \end{array}$$

(8) $$\begin{array}{l} {\hat{x}}_{k\;+\;1}=A{\hat{x}}_{k}+B{\hat{u}}_{k}+A{\left(\Sigma_{k}^{-1}+C^{T}N^{-1}C-\gamma^{-2}Q_{k}\right)}^{-1}\\ \;\cdot \;\left\{\gamma^{-2}Q_{k}{\hat{x}}_{k}+C^{T}N^{-1}\left(y_{k}-C{\hat{x}}_{k}\right)\right\}. \end{array}$$

The estimated disturbance diverges to infinity if the level of disturbance attenuation γ is close to zero. Thus, the level of γ cannot be chosen freely and must satisfy the following constraints:

(9) $$M_{k\;+\;1}^{-1}-\gamma^{-2}DD^{T}>0\text{ and }\Sigma_{k}^{-1}-\gamma^{-2}Q_{k}>0.$$

The strong concavity condition is given by

(10) $${\tilde{\Sigma }}_{k\;+\;1}^{-1}-\gamma^{-2}M_{k\;+\;1}>0\text{ or }\Sigma_{k}^{-1}-\gamma^{-2}{\tilde{M}}_{k}>0,$$

where

$$\begin{array}{l} {\tilde{M}}_{k}=A^{T}{\left(M_{k\;+\;1}^{-1}-\gamma^{-2}DD^{T}\right)}^{-1}A+Q_{k},{\tilde{M}}_{N}=Q_{N},\\ {\tilde{\Sigma }}_{k\;+\;1}=A{\left(\Sigma_{k}^{-1}-\gamma^{-2}Q_{k}\right)}^{-1}A^{T}+DD^{T},{\tilde{\Sigma }}_{1}=Q_{0}^{-1}. \end{array}$$

The constraints above can be translated into equivalent conditions on the spectral radius (i.e., the maximum of the absolute values of the eigenvalues) because the spectrum radius is equal to the norms of **M**_*k* + 1_ **DD**^*T*^, **Σ**_*k*_ **Q**_*k*_, $\tilde{\Sigma }$_*k* + 1_ **M**_*k* + 1_, and **Σ**_*k*_ $\tilde{M}$_*k*_. Thus, Equations (9) and (10) require the following conditions be satisfied: ρ(**M**_*k* + 1_**DD**^*T*^) < γ^2^, ρ(**Σ**_*k*_ **Q**_*k*_) < γ^2^, and ρ($\tilde{\Sigma }$_*k* + 1_ **M**_*k* + 1_) < γ^2^ or ρ(**Σ**_*k*_ $\tilde{M}$_*k*_) < γ^2^, where ρ is the kernel of the spectral radius.

### Application to a sensorimotor system

We applied the MMFC approach to a sensorimotor system. The dynamics model is based on previous studies (Todorov, 2005; Izawa and Shadmehr, 2008; Izawa et al., 2008; Braun et al., 2009). The dynamics were simulated with uncertainties, represented by force fields: a velocity-dependent force field (VF) and a divergent force field (DF), representing stable and unstable environments, respectively (Franklin et al., 2003a,b; Osu et al., 2003). We also designed both optimal and mini-max feedback controllers for the problem and compared their performances.

#### Sensorimotor system

***Dynamics model.*** We modeled a movement with two degrees of freedom, such as multi-joint flexion and extension of the shoulder and elbow joints, as cursor movements on a screen, described by shifting the position **p**(*t*) = [*x*(*t*), *y*(*t*)]^*T*^ to designated targets p^*^ = [x^*^, y^*^]^*T*^:

(11) $$m\overset{..}{p}(t)=f(t)-b\dot{p}(t),$$

where *m* and *b* are the end-point mass and viscosity, respectively, and are set equal to *m* = 1.0 (kg) and *b* = 10 (Ns/m). The combined action of all muscles is represented by the force vector **f**(*t*) ∈ *R*^2^ acting on the hand. The motor command **u**(*t*) ∈ *R*^2^ is transformed into the force **f**(*t*) by adding control-dependent multiplicative noise and by applying a simplified first-order muscle-like low-pass filter of the following form:

(12) $$\dot{f}(t)=\frac{\left(I+\sigma_{u}\varepsilon (t))u(t)-f(t\right)}{\tau },$$

with time constant, τ = 0.05 (s). The motor command **u**(*t*) is disturbed by signal-dependent multiplicative noise that exists in the neural system (Matthews, 1996), and plays an important role in motor planning (Harris and Wolpert, 1998). The signal-dependent noise (SDN) is given by the Gaussian white noise **ε**(*t*) ~ *N*(**0**, **I**) and the magnitude **σ**_**u**_ is set equal to 0.5.

***Observation model.*** In our model, the state variables cannot be observed directly. The sensory output **y**(*t*) ∈ *R*^8^ is the position, velocity, force, and target position disturbed by sensory noise, and is given by:

(13) $$y(t)=\left[\begin{array}{c} p(t)\\ \dot{p}(t)\\ f(t)\\ p^{*} \end{array}\right]+\sigma_{y}v(t),$$

where **v**(*t*) ∈ *R*^8^ and **σ**_**y**_ ∈ *R*^8 × 8^ are the Gaussian white noise **v**(*t*) ~ *N*(**0**, **I**) and the diagonal matrix defined by **σ**_**y**_ = *diag*([0.02*c*, 0.02*c*, 0.2*c*, 0.2*c*, *c*, *c*, 0, 0]), respectively. Here, *c* is the scaling parameter, equal to the SDNs: i.e., *c* = **σ**_**u**_ = 0.5, similar to a previous study (Todorov, 2005). The task is to move the hand from the starting position **p**(0) = [0, 0]^*T*^ to the target position p^*^, which is located at a distance of 25 cm, and to stop at the terminal period between 600 and 700 ms, in accordance with experiments (Franklin et al., 2003a,b; Osu et al., 2003).

#### Environmental uncertainty

**Figure 2:**
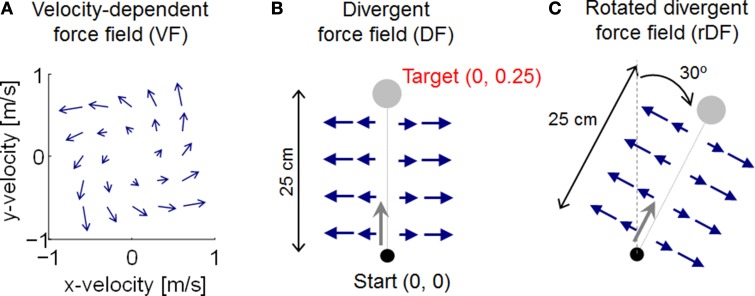
**Illustration of force field dynamics**. **(A)** Velocity-dependent force field (VF). **(B)** Divergent force field (DF). **(C)** Rotated divergent force field (rDF). The target and applied force are rotated 30° in the clockwise direction.

We assumed two different types of force field as uncertainty environments, VF and DF. The force fields exert a force **F**_*ext*_(*t*) ∈ *R*^2^ on the hand. The force generated by the VF is

(14) $$F_{ext}(t)=F_{VF}\dot{p}(t),F_{VF}=\alpha \left[\begin{array}{cc} 13 & -18\\ 18 & 13 \end{array}\right],$$

where α is a scaling parameter, set equal to 0.1 to generate effective perturbation for the trajectory. When reaching forward, the force is directed forward and to the left, as the velocity along the *y*-axis is increased (Figure 2A). DF produces a negative elastic force perpendicular to the target directions, with a value of zero along the *y*-axis: i.e., no force is applied when the path of the hand follows the *y*-axis, but the hand is pushed away whenever it deviates from the *y*-axis (Figure 2B). DF teaches subjects to move in a straight line, but to show no after-effects on the removal of the field. The task is achieved by increasing the stiffness of the arm, but only in the direction of maximum instability (Figure 1A). The force generated by DF is described by

(15) $$F_{ext}(t)=F_{DF}p(t),F_{DF}=\beta \left[\begin{array}{cc} 1 & 0\\ 0 & 0 \end{array}\right],$$

where β is a scaling parameter, set equal to 100 to generate effective perturbation for the trajectory. Although end-point trajectories were almost straight without external dynamics, the initial movement direction varied slightly from trial to trial, due to motor output variability (Burdet et al., 2001). Thus, because DF produces an unstable interaction with the arm to amplify such variation by pushing the hand with a force proportional to the deviation from the *y*-axis, the initial trials in DF exhibited unstable behavior, diverging widely to the right or left of the *y*-axis. We also examined the additional DF case of a rotated divergent force field (rDF), which is necessary to reach a rotated position (Figure 2C). The exerted force is also rotated. If the target is realigned at an angle θ in the clockwise direction, the force is then given by

(16) $$F_{DF}=\beta \;\left[\begin{array}{cc} \cos^{2}\theta & \sin\theta \cos\theta \\ \sin\theta \cos\theta & \sin^{2}\theta \end{array}\right].$$

In this study, the rotational angle θ was set equal to 30°. With these force fields, the dynamic uncertainties can be expressed as follows:

$$\Delta_{A}=\left[\begin{array}{ccc} 0_{4\times 2} & 0_{4\times 2} & 0_{4\times 6}\\ 0_{2\times 2} & F_{VF} & 0_{2\times 6}\\ 0_{2\times 2} & 0_{2\times 2} & 0_{2\times 6} \end{array}\right]\text{ or }\Delta_{A}=\left[\begin{array}{ccc} 0_{4\times 2} & 0_{4\times 2} & 0_{4\times 6}\\ F_{DF} & 0_{2\times 2} & 0_{2\times 6}\\ 0_{2\times 2} & 0_{2\times 2} & 0_{2\times 6} \end{array}\right].$$

In both cases, the environmental uncertainties do not depend on the motor command; however, the motor command is disturbed by the SDN. Thus, the uncertainty of the motor command is represented by Δ_**B**_ = **σ**_**u**_ · **B** · **ε**(*t*).

#### Controller design

We carried out numerical simulations using both OFC and MMFC to compare their performances. In our simulations, the dynamics model was rewritten as a discrete-time system, using a state-space formulation:

(17) $$x_{k\;+\;1}=Ax_{k}+B\left(I+\sigma_{u}\varepsilon_{k}\right)u_{k},$$

(18) $$y_{k}=Cx_{k}+\sigma_{y}v_{k},$$

where **x**_*k*_ ∈ *R*^8^ is a state-space vector at time step *k*, defined by **x**_*k*_ = [**p**^*T*^_*k*_, **Ω**^*T*^_*k*_, **f**^*T*^_*k*_, p^* *T*^]^*T*^. The matrices describing the system, **A** ∈ *R*^8 ×8^, **B** ∈ *R*^8 × 2^, and **C** ∈ *R*^8 × 8^, are expressed as follows:

$$\begin{array}{l} A=\left[\begin{array}{cccc} I_{2\times 2} & \Delta \cdot I_{2\times 2} & 0_{2\times 2} & 0_{2\times 2}\\ 0_{2\times 2} & I_{2\times 2} & \Delta /m\;\cdot \;I_{2\times 2} & 0_{2\times 2}\\ 0_{2\times 2} & 0_{2\times 2} & (1-\Delta /\tau )\;\cdot \;I_{2\times 2} & 0_{2\times 2}\\ 0_{2\times 2} & 0_{2\times 2} & 0_{2\times 2} & I_{2\times 2} \end{array}\right],\\ B=\left[\begin{array}{c} 0_{2\times 2}\\ 0_{2\times 2}\\ \Delta /\tau \cdot I_{2\times 2}\\ 0_{2\times 2} \end{array}\right],C=I, \end{array}$$

where Δ is a single time step of the simulation, set equal to Δ = 0.005 s.

In these simulations, we assumed two types of condition: (i) with structural uncertainty and (ii) without structural uncertainty. Under the condition with structural uncertainty, the system matrix **A** of the state-space Equation (3) is not equal to the actual dynamics including the force field. In contrast, under the condition without structural uncertainty, the force field dynamics are completely represented in the internal model. Thus, the system matrix **A** in Equation (3) is replaced by **A** + Δ_**A**_.

***Optimal feedback controller.*** An optimal feedback controller also generates motor commands, thus forming state feedback, as in Equation (6). The feedback gain is computed to minimize the following cost function:

(19) $$\begin{array}{l} J(u)=\sum_{k\;=\;N_{s}\;+\;1}^{N}\left(w_{p}^{2}{\left\|p_{k}-p^{*}\right\|}^{2}+w_{v}^{2}{\left\|{\dot{p}}_{k}\right\|}^{2}+w_{f}^{2}{\left\|f_{k}\right\|}^{2}\right)\\ \;+\sum_{k\;=\;1}^{N\;-\;1}{\left\|u_{k}\right\|}^{2}, \end{array}$$

where *w*_*p*_, *w*_*v*_, and *w*_*f*_ are the cost weights of the end-point position, velocity, and force, with the assigned values, *w*_*p*_ = 10^4^, *w*_*v*_ = 10^3^, and *w*_*f*_ = 10^2^, respectively, to achieve the reaching task adequately without external dynamics, i.e., null force field (NF). In addition, the terminal cost is defined to evaluate the states between *N*_*s*_ = 0.6/Δ and *N* = 0.7/Δ steps. Thus, the cost function requires the expected state to be stabilized at close to the target in the terminal period (*N_*s*_* < *k* < *N*). The feedback gain is determined by

(20) $$L_{k}={\left(B^{T}S_{k\;+\;1}B+I\right)}^{-1}B^{T}S_{k\;+\;1}A,$$

where **S**_*k* + 1_ is found by solving the Riccati equation

$$S_{k}=A^{T}S_{k\;+\;1}(A-B^{T}L_{k})+Q_{k}\text{ with }S_{N}=Q_{N},$$

where **Q**_*k*_ ∈ *R*^8 × 8^ is the task cost matrix, given by **Q**_*k*_ = **q**^*T*^_*k*_**q**_*k*_, where

$$\begin{array}{l} q_{k}=\left[\begin{array}{cccc} w_{p}\;\cdot \;I_{2\times 2} & 0_{2\times 2} & 0_{2\times 2} & -w_{p}\;\cdot \;I_{2\times 2}\\ 0_{2\times 2} & w_{v}\;\cdot \;I_{2\times 2} & 0_{2\times 2} & 0_{2\times 2}\\ 0_{2\times 2} & 0_{2\times 2} & w_{f}\;\cdot \;I_{2\times 2} & 0_{2\times 2}\\ 0_{2\times 2} & 0_{2\times 2} & 0_{2\times 2} & 0_{2\times 2} \end{array}\right]\;(k>N_{s}),\\ \text{or }q_{k}=0\;(k\leq N_{s}). \end{array}$$

The cost weights are also used in the mini-max feedback controller design, and tuned to accomplish the tasks for all conditions in the MMFC. The state of the system is estimated from noisy observation using Kalman filtering and is expressed as follows:

(21) $${\hat{x}}_{k\;+\;1}=A{\hat{x}}_{k}+Bu_{k}+K_{k}(y_{k}-C{\hat{x}}_{k}),$$

where **K**_*k*_ ∈ *R*^8× 8^ is the Kalman gain: i.e., a function of the uncertainty of the estimated state and the measurement noise. We adapted a standard technique to calculate the gain, as follows:

(22) $$K_{k}=P_{k\;|\;k\;-\;1}C^{T}{\left(CP_{k\;|\;k\;-\;1}C^{T}+\sigma_{y}\sigma_{y}^{T}\right)}^{-1},$$

where **P**_*k*|*k* − 1_ ∈ *R*^8×8^ is the predicted accuracy of the state estimation and is given by

$$\begin{array}{l} \;P_{k|k\;-\;1}=AP_{k\;-\;1|k\;-\;1}A^{T}+(B\sigma_{u}u_{k}){\left(B\sigma_{u}u_{k}\right)}^{T}\\ \text{with }P_{k|k}=(I-K_{k}C)P_{k|k\;-\;1}. \end{array}$$

The Kalman gain is computed concurrently at each time step in the simulation, starting with the initial condition **P**_0|0_ = 10^−3^ × **I**.

***Mini-max feedback controller.*** To apply the MMFC approach, uncertainty must be modeled as familiarity with itself. Thus, we represent the familiarity by the matrices **D**_*a*_ ∈ *R*^8 ×8^, **D**_*b*_ ∈ *R*^8 × 8^, **D**_*c*_ ∈ *R*^8 × 8^, and **D**_*y*_ ∈ *R*^8 × 8^, given by **D**_*a*_ = κ Δ_**A**_, **D**_*b*_ = λ **σ**_**u**_**B**, **D**_*c*_ = **0**, and **D**_*y*_ = λ **σ**_*y*_, where κ and λ are the scaling parameters of familiarity. The parameter κ was set to a range of a closed interval [0, 1]. When the force field dynamics cannot be predicted—i.e., **D**_*a*_ = **0** (κ = 0)–the structural uncertainty is not modeled. When the force field dynamics are modeled completely as the structural uncertainty, **D**_*a*_ = Δ_**A**_ (κ = 1). The controller is then designed to maximize the effect of the dynamics as the worst-case assumption. In addition, **D**_*b*_ and **D**_*y*_ must be sufficiently large to exceed the maximum value of distribution, and hence the scaling parameter λ is set to λ = 5. This seems sufficient for the disturbances, because the SDN and sensory noise have a standard Gaussian white noise distribution.

Matrices representing the regulated outputs **E**_*a*_ ∈ *R*^8 × 8^, **E**_*b*_ ∈ *R*^8 × 8^, and **E**_*c*_ ∈ *R*^8 × 8^ were given to satisfy the assumption **G**^*T*^**G** = **I** and **H**^*T*^**G** = **0** by:

(23) $$E_{a}=\{\begin{array}{c} q_{k}\;(k>N_{s})\;\\ I\;(k\leq N_{s})\; \end{array},E_{b}=I,E_{c}=0.$$

The terminal cost matrix **Q**_*N*_ has already been defined in the optimal feedback controller design, and the initial error cost **Q**_0_ ∈ *R*^8 × 8^ is defined as **Q**_0_ = **P**_0|0_. Finally, the disturbance attenuation level γ is set equal to 10^7^ to satisfy Equations (9) and (10).

## Results

We performed numerical simulations of point-mass reaching movement in different types of force fields–VF, DF, and rDF–using OFC and MMFC. The simulations were carried out 100 times for each case.

### Comparison of trajectories

**Figure 3:**
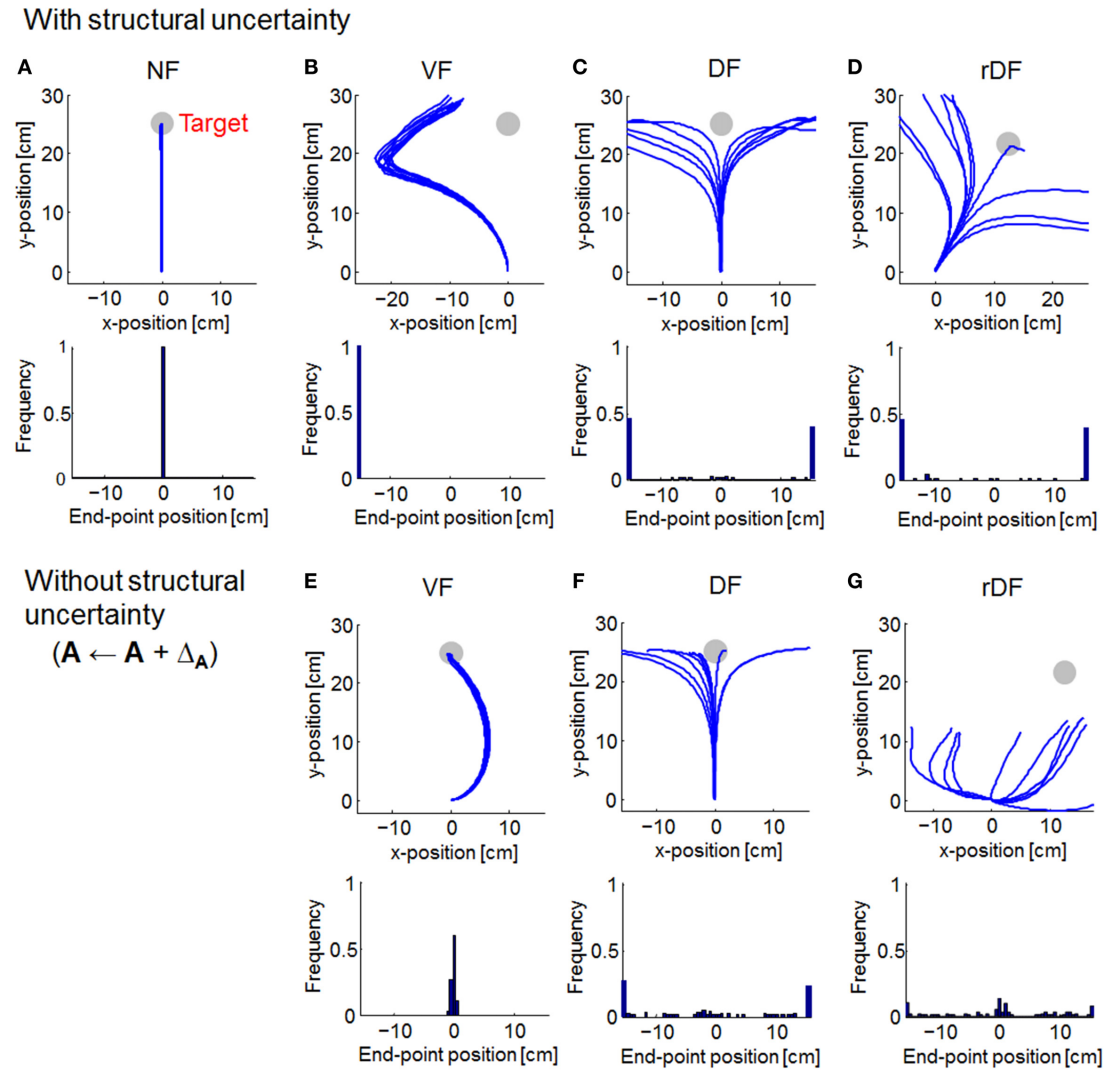
**Simulated trajectories of optimal feedback control (OFC)**. Solid lines indicate the simulated trajectories for 10 randomly selected simulations. Gray circles denote the targets. Histograms at the bottom of the trajectories show distributions of the end-point positions (based on curvature > 0.03 mm^−1^). **(A)** Null force field (NF) condition. **(B–D)** With structural uncertainty of force fields. **(E–G)** Without structural uncertainty of force fields.

We compared the trajectories of OFC and MMFC. Then, the end-point distributions were computed from the lateral distances of the target direction (based on curvature > 0.03 mm^−1^), following a previous report (Osu et al., 2003). The trajectories were almost straight lines for OFC in NF (Figure 3A). However, under conditions of a force field, reaching the target was difficult. In VF, the trajectories curved to the left (Figure 3B). In DF and rDF, the trajectories diverged to the left and right in accordance with the directions of the targets (Figures 3C,D). When the force field dynamics were modeled internally, i.e., **A** ← **A** + Δ_**A**_, the trajectories in VF came close to the targets with a curve; however, the trajectories of DF and rDF did not achieve their targets (Figures 3E–G). Under the rDF condition, in particular, some trajectories could not aim toward the target even immediately after the onset of movement. The behavior difference from DF was caused by cross talk in the coordinates. In DF, deviance on the *x*-axis was independent on the hand position on *y*-axis, because the diagonal components of the feedback gain were zero. In rDF, conversely, the lateral deviancy affected the vertical distance between the target hand positions through the feedback gain, and the task required more motor effort to reach the same distance to DF because each actuator acted on only the *x*- or *y*-axis.

**Figure 4:**
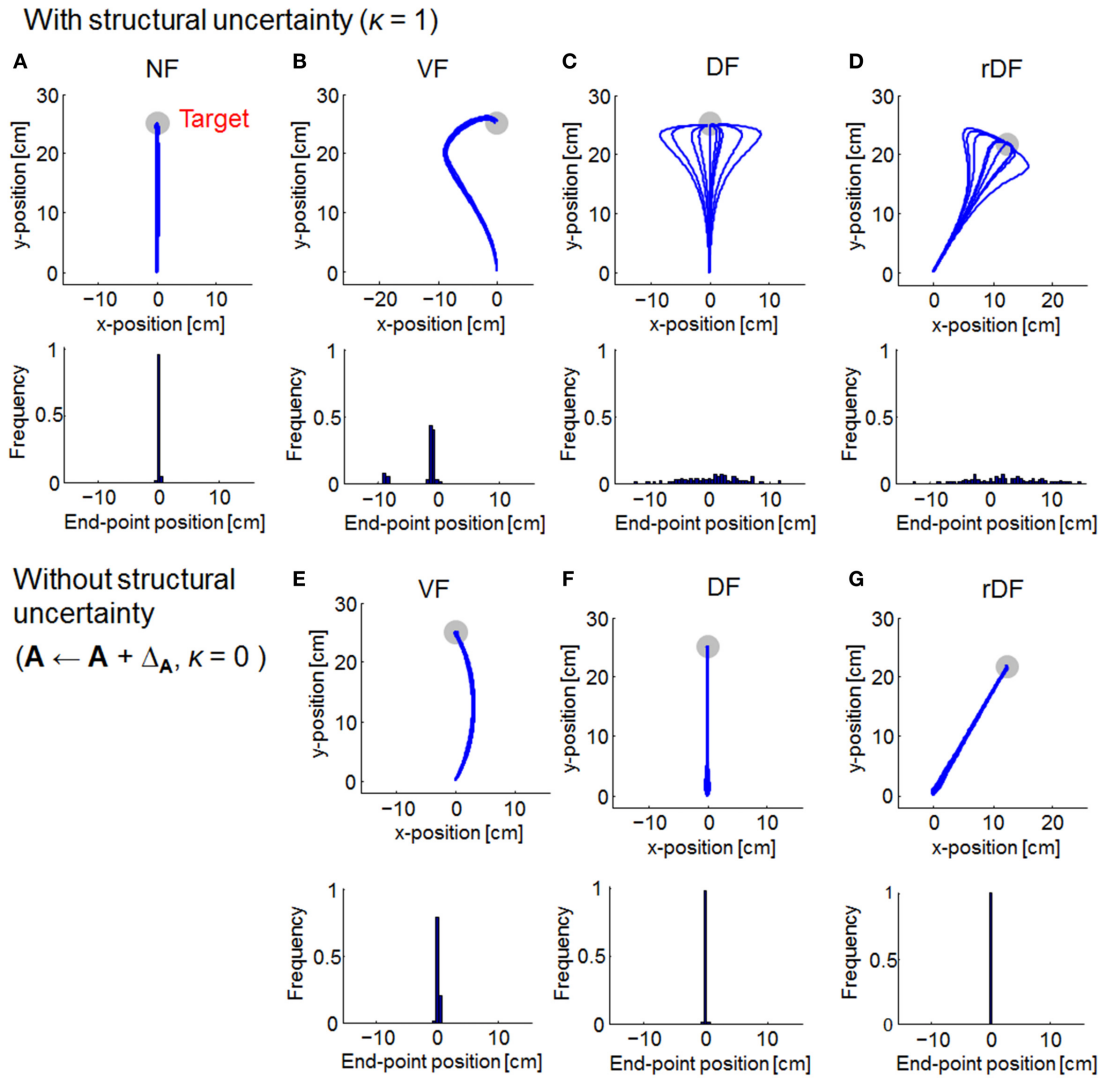
**Simulated trajectories of MMFC for 10 randomly selected simulations**. The format is the same as in Figure 3. In **(A–D)**, the parameter of familiarity with the uncertainty κ is set equal to 1. In **(E–G)**, the familiarity parameter κ is set equal to 0.

As with OFC, the trajectories of MMFC were almost straight lines in NF (Figure 4A). In VF, although the trajectories curved gradually after the onset of movement, they turned suddenly toward the target, even when the force field dynamics was not completely known (Figure 4B). In DF and rDF, even if the trajectories had diverged after the onset of movement, they finally converged to the target (Figures 4C,D). These trajectories were similar to those obtained from the results of initial trials, during adaptation to the same types of dynamics, in human experiments (Osu et al., 2003). However, in VF without structural uncertainty of force fields, i.e., **A** ← **A** + Δ_**A**_ and κ = 0, the trajectories curved slightly to the right direction and achieved the target (Figure 4E). Subsequently, the trajectories were straight lines in DF and rDF (Figures 4F,G).

**Figure 5:**
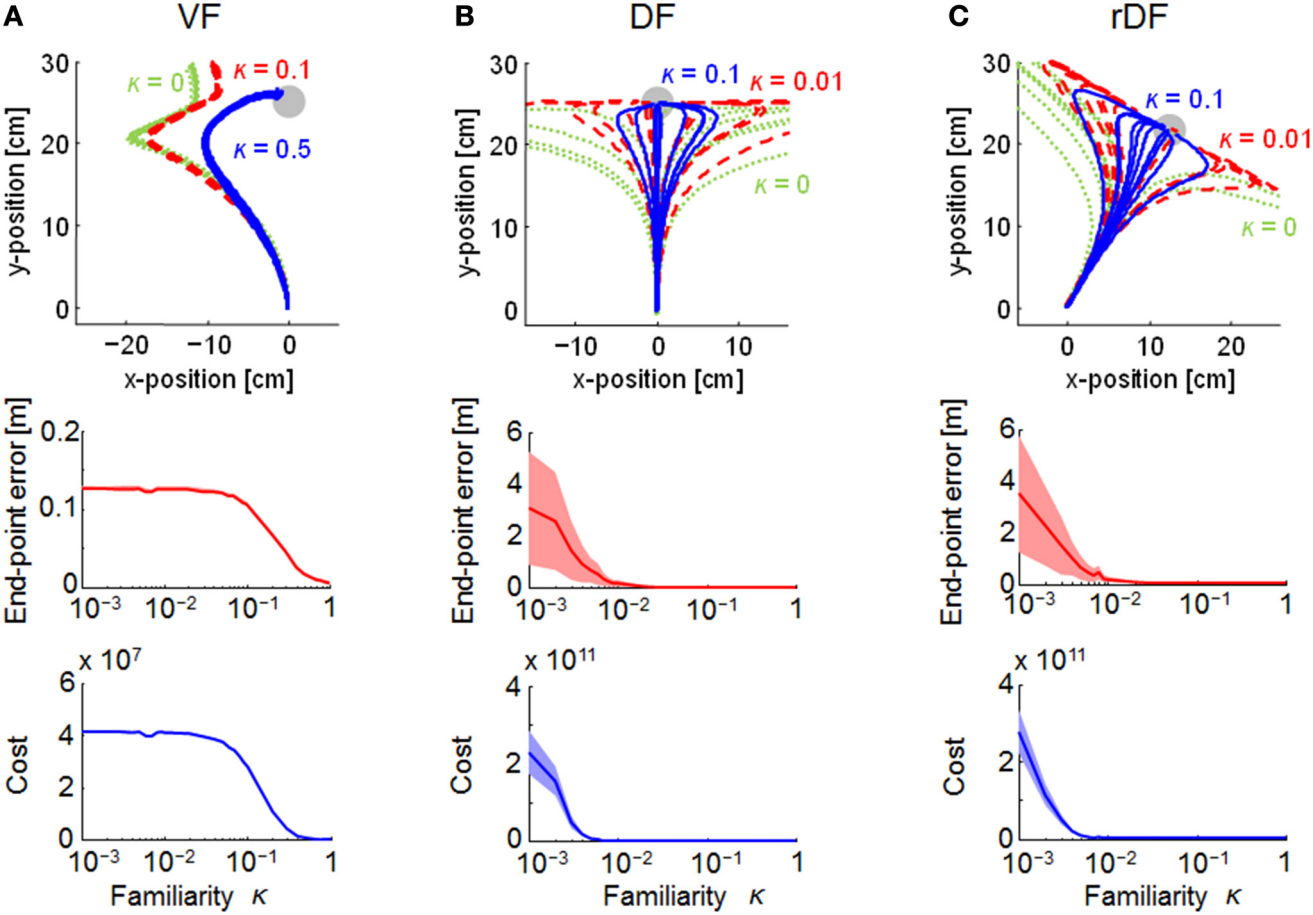
**Effect of the range of structural uncertainty on the trajectories in MMFC**. Green dotted, red dashed, and blue solid lines indicate lower, middle, and higher uncertainties, respectively, given by the familiarity parameter κ. The top row indicates the end-point trajectories. The middle and bottom rows are single logarithmic plots of the terminal end-point error (mean ± *SD*) and the mean quadratic costs and the 95% confidence intervals (CIs). The CIs were estimated using a bootstrapping procedure with re-sampling 10,000 times. **(A–C)** represent VF, DF, and rDF conditions, respectively.

The familiarity parameter κ (0 ≤ κ ≤ 1) affects the performance of MMFC directly, because the structural uncertainty of the force fields was not reflected in the motor control when κ = 0. Thus, we evaluated the effect on the trajectories (Figure 5). When κ = 0, the trajectories could not reach the targets in all conditions, and those of DF and rDF diverged. With the increase in the parameter κ, the trajectories were close to the targets under all conditions. The variability of the trajectories as well as the end-point errors were decreased in DF and rDF. In addition, the quadratic costs, given by Equation (19), decreased to a slightly greater degree than those of the end-point errors, and the performances were saturated at around κ = 0.5 in VF, and κ = 0.01 in DF and rDF.

### Feedback gain geometries

There are mathematical difficulties in incorporating the impedance generated by non-linear muscular properties with a feedback control law. However, several studies have provided evidence that sensorimotor control systems can and do regulate feedback gains for impedance control (Franklin et al., 2007; Krutky et al., 2010; Franklin and Wolpert, 2011). Although the impedance is not actually equal to the feedback gains computed by OFC or MMFC, the gains must contribute to the modulation of impedance. Thus, we computed sensory feedback gains, transferring sensory feedback errors to the motor command as products of the state feedback and filter gains. The sensory feedback gains for OFC and MMFC were then given by products of the state feedback and filter gains, as **L**_*k*_ · **K**_*k*_ and **L**_*k*_ · **A**(**Σ**_*k*_ + **C**^*T*^**N**^−1^*C* − γ^−2^**Q**_*k*_)^−1^**C**^*T*^**N**^−1^, respectively. We visualized the patterns of the positional gain at the midpoints of the movement time (350 ms) as ellipses, similar to the stiffness ellipses used previously (Burdet et al., 2001; Franklin et al., 2003a, 2007; Ueyama and Miyashita, 2014). The orientation, shape, and size of the ellipse are obtained by singular value decomposition of the positional gain matrix.

**Figure 6:**
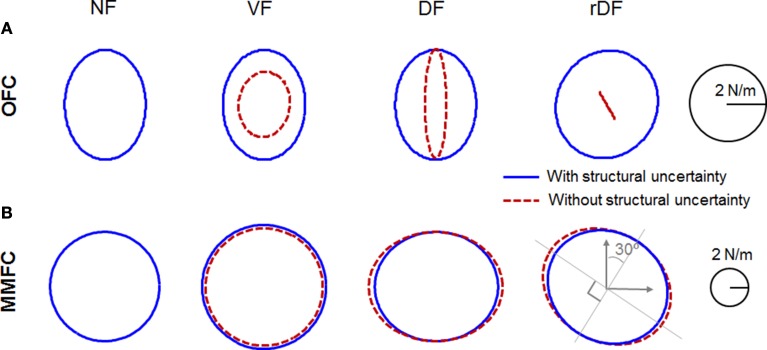
**Positional sensory feedback gain geometries at the midpoint of the movements in Figures 3, 4**. Red dotted and blue solid lines indicate the condition with uncertainty and without uncertainty, respectively. Each row indicates NF, VF, DF, and rDF conditions from the left. **(A)** Ellipses for OFC. **(B)** Ellipses for MMFC.

In NF, the gain of OFC was a vertically long ellipse (Figure 6A). In VF and DF of OFC, the gains with structural uncertainty were quite similar to the gain in NF. However, the gain in VF without structural uncertainty was rather small, and varied by ~4° in the clockwise direction; that of DF decreased in a lateral direction. In rDF, the gain with structural uncertainty was directed to the target at 30° in a clockwise direction. However, the gain without structural uncertainty was diminished and directed to −60° in a clockwise direction. As mentioned in Section Comparison of Trajectories, the lateral deviancy and target directed movement influenced each other through feedback gain. In particular, the *y*-axis movement was more dependent on the *x*-position than the *y*-position. Thus, the task required complicated cooperative action, and the gain geometry was squashed. However, the gain of mini-max feedback control in NF was a true circle, and larger than that of OFC (Figure 6B). The gains in VF also indicated true circles, even if they were larger than those of NF. In DF and rDF, the gains were tuned by the force field, according to the direction of instability, as in the experimental measurements of stiffness (Franklin et al., 2007). In DF, only the lateral axes of the gains were expanded, although the anteroposterior axes were the same as those of NF. In rDF, the gains were similar to the 30° rotations of those in DF. The gain without structural uncertainty of VF was a little smaller than that with uncertainty dynamics. In contrast, the gains in DF and rDF also increased toward the unstable directions, as in the conditions with structural uncertainty.

## Discussion

In this study, MMFC is presented as an extension of OFC for use as a robust control technique. This method uses time-varying feedback control for estimated states, including worst-case disturbances expected by familiarity with novel dynamics. The uncertainties of dynamics and noise are defined as disturbances in accordance with a robust control theory. In previous research, the uncertainties were assumed to have a Gaussian distribution (Bays and Wolpert, 2007; Izawa and Shadmehr, 2008; Crevecoeur et al., 2010); however, it seems unlikely that real-world uncertainties would do so. Accordingly, we modeled the uncertainties of environmental dynamics as structural uncertainties, using the robust control design. The computational method seems adequate, because the central nervous system can minimize the uncertainty of sensory input in two ways: by combining multiple sensory signals with prior knowledge to refine sensory estimates, and by predictive filtering of sensory input to remove less informative components of the signal (Bays and Wolpert, 2007). The simulation results indicated greater performance for environmental dynamics of force fields in terms of robustness and stability, and also reproduced behavioral characteristics. Thus, we consider that MMFC could predict motor behavior in the presence of structural uncertainty, and explain the early process of motor adaptation because it was able to predict a behavior, and achieve the task without environmental information. Furthermore, the feedback gain was increased in unstable directions like the stiffness modulation of a multi-joint arm in arm-reaching movements with unstable dynamics. This suggests that the brain modulates optimal stiffness to obtain efficient robustness, overwhelming the instabilities of the environmental dynamics. Moreover, a recent study suggested that reflex gains (feedback gains) are modulated by the accumulated evidence in support of an evolving decision before the onset of movement (Selen et al., 2012). This seems to support our theory, in that the feedback gains are determined according to the uncertainty of the movement in the motor planning phase before the onset of movement.

**Figure 7:**
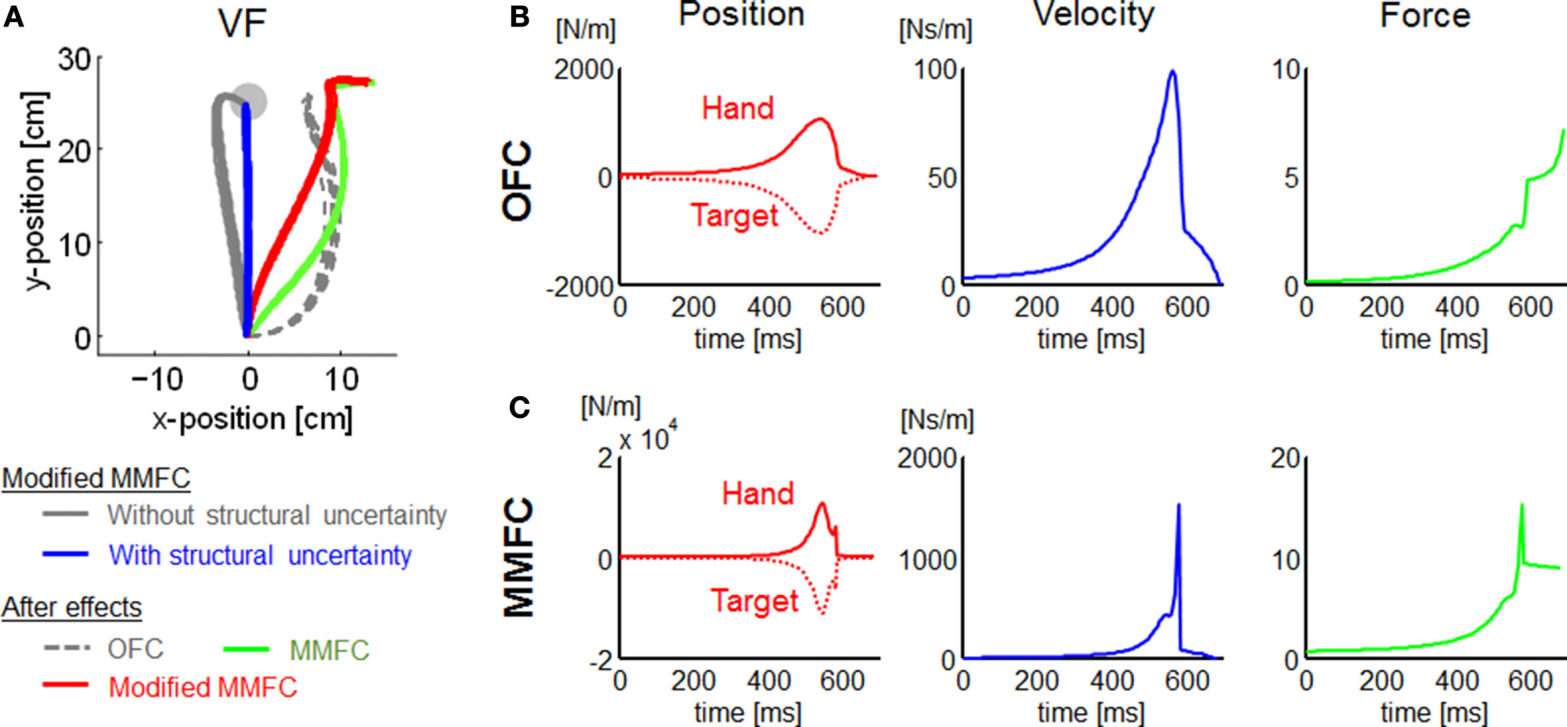
**Additional simulations for VF conditions, and state feedback gain profiles for NF conditions. (A)** Simulated trajectories of the modified MMFC for VF conditions, and the after-effects of each model. **(B)** Gains for OFC. **(C)** Gains for MMFC. In **(B,C)**, each row indicates position, velocity, and force gains from the left. With the position gains, the solid and dotted lines indicate the hand and target position gains, respectively, which are opposites in sign. Note that γ = 10^8^ to satisfy Equations (9) and (10).

The trajectories in VF were somewhat different between our simulations and experimental measurements (Osu et al., 2003). Our simulations of the OFC and MMFC models could not predict the straight trajectory observed in the human study. The result may give the false impression that a trajectory control strategy to reduce motor effort requires a distinct deviation from the nominal straight line. However, the theoretical framework such as OFC actually may not be incompatible with the trajectory control by a cost function that trades off the discordant requirements of target accuracy, motor effort, and kinematic invariance in an acceleration-dependent force field (Mistry et al., 2013). This approach could be considered a MMFC representing the deviation from the straight line with a disturbance. During the period of movement (*k* ≤ *N*_*s*_), we defined the regulated output matrix **E**_*a*_ as an identity matrix to generalize the MMFC model for motor adaptation problems. However, it was assumed that **E**_*a*_ transfers the state vector into a disturbance, which is determined by the kinematic constraints, bootstrapping the process of exploration and learning. The kinematic constraints appear reasonable to improve the task, particularly in the early phase of motor adaptation. Thus, we carried out extra VF simulations to examine this assumption. Then we modified the MMFC to replace **E**_*a*_ (*k* ≤ *N*_*s*_) in Equation (23) with 100 · *diag*([1, 0, 1, 0, 0, 0, 0, 0]) as a kinematic constraint penalizing lateral deviances of the position and velocity (Mistry et al., 2013). Unsurprisingly, the modified MMFC resulted in trajectories that were close to linear (Figure 7A). Furthermore, the modified MMFC showed closer trajectories to the linear behavior than other models. These results suggest that kinematic constraints may be applied to determine an MMFC with environmental dynamics to ensure kinematic invariance.

It has been suggested that a cost function should be modulated to increase the ratio of the energy cost, according to the uncertainty of the internal model (Crevecoeur et al., 2010), and standard forms for quantifying cost may not be sufficient to accurately examine whether human motor behavior abides by optimality principles (Berniker et al., 2013). In the model proposed here, the terms expressing the familiarity with the uncertainty are related to the cost values. That is, the cost function is indirectly modulated via the uncertainty of the internal model, which itself may also be reflected in the nervous system's use of impedance control to change the dynamic properties of the body (Burdet et al., 2001; Takahashi et al., 2001; Lametti et al., 2007; Mitrovic et al., 2010). These studies support our proposed model.

### Limitations of this model

In our simulation results, the changes in the gain in the direction of instability for the DF and rDF of MMFC model are fairly small compared to the magnitude of the experimental measurement (Franklin et al., 2007). This non-conformity may be caused by differences between actual and modeled muscle dynamics. In this study, arm dynamics was simplified as a linear point-mass model. However, the biological arm movement is actually induced by many muscles with non-linear dynamics. The muscle action forms limb stiffness geometry depending on task requirements. Our model did not reflect actual muscle dynamics. Especially, passive muscle mechanisms were not considered in the model. Even when the muscle is relaxed (the activation level is decreased), the active force disappears and the resting length is restored by the passive force (Huxley and Hanson, 1954). Then, limb stiffness is retained during maintained posture without muscle contraction, and the magnitude is not small, compared to muscle contraction effects on that (Osu and Gomi, 1999; Shin et al., 2009). Because the passive limb stiffness acts to inhibit the intended movement, agonist muscles are required to generate active force overwhelming the passive force retaining the posture to initiate movement. Thus, actual limb stiffness may be much higher than that in our simulations.

However, the feedback gain magnitude was small compared to the proprioceptive and visual feedback responses measured in human subjects (Bennett, 1994; Dimitriou et al., 2013). The difference between our simulation and the proprioceptive feedback response (the reflex response) may be attributable to the rigidity of the muscle model, analogous to the magnitude of stiffness modulation. The visual feedback gains measured in humans were purposed not to fall into the sensory feedback gain but the state feedback gain (Dimitriou et al., 2013). Moreover, the response was computed from a time window of 180–230 ms after perturbation onset, and it was not considered how the state estimation was updated for feedback latency. In fact, although the magnitude did depend directly on the cost weights and our model did not separate the visual feedback response from other feedback, our simulations for OFC and MMFC models showed sufficiently large feedback gains, exceed the feedback response reported in humans (Figures 7B,C). It has been suggested that the feedback gains show different time profiles. The visual feedback gain showed peaking at the middle of the movement and dropping rapidly at the movement end (Liu and Todorov, 2007; Dimitriou et al., 2013). In contrast, intrinsic feedback gain, measured as stiffness, showed a contrary profile, peaking at the movement onset and end, and dropping in the middle of the movement (Gomi and Kawato, 1997; Ueyama and Miyashita, 2014).

### Other models

Although an adaptation algorithm for uncertain dynamics has been proposed (Franklin et al., 2008), it is based on a feedback-error-learning strategy and requires a desired trajectory (Kawato, 1996; Ueyama and Miyashita, 2014). Thus, the adaptation process and motor planning of the desired trajectory must be considered separately and handled as different problems. In contrast, a MMFC can deal with both issues in the same context, as does OFC.

Friston raised the question of differences between internal models in motor control and perceptual inference in OFC, and suggested that active inference, a corollary of the free-energy principle, reduces to simply suppressing proprioceptive prediction errors (Friston, 2011). Moreover, it has been reported that active inference could acquire complex and adaptive behaviors using a free-energy formulation of perception (Friston et al., 2009), and generate movement trajectories shown to be remarkably robust to perturbations on a limb (Friston et al., 2010). In active inference, the cost function is absorbed into prior beliefs about state transitions and terminal states. Thus, active inference seems attractive as a means of recognizing biological optimization mechanisms, because OFC and MMFC have many free parameters (e.g., cost function and terminal time) that intricately affect the behavior. However, the behavior of active inference seems to be influenced by the estimated probability (i.e., prior assumption of noise and uncertainty) as a substitute for the definition of cost function. We consider that active inference and OFC are not mutually exclusive, and that the free-energy principle is just a “principle” that could unify motor control theories, based on the optimization of a cost. Although the free-energy principle has not been derived from empirical evidence, it can predict neurobiological implementation from the perspective of functional anatomy (Friston et al., 2012). For motor control studies, therefore, the free-energy principle seems to be a useful tool to connect the computational level to the hardware level.

Recently, behavioral studies have focused on understanding how uncertainty, or risk, is represented in motor control tasks, as well as in economic behaviors (Trommershäuser et al., 2008). Violations of risk neutrality have been reported various motor control tasks. For example, subjects exhibited risk-seeking behavior in a pointing task, because they systematically underestimated small probabilities and overestimated large probabilities (Wu et al., 2009). In addition, subjects exhibited risk-average behavior in a motor task that required them to control a Brownian particle with different levels of noise, which is consistent with the notion of a trade-off between the mean and the variance of movement cost (Nagengast et al., 2010). Moreover, it has also been suggested that the sensitivity of the risk is an important factor in motor tasks with speed/accuracy trade-offs (Nagengast et al., 2011). In contrast, when the uncertainty is assumed to have a Gaussian distribution and an exponential-quadratic error criterion, such as the expected unity function describing risk sensitivity, is used as the cost function, the MMFC problem is identified with the risk-sensitive optimal control problem of optimizing the exponential-quadratic error criterion (Speyer et al., 1992). Furthermore, an equivalence has already been established between a deterministic robust control that achieves a prescribed bound on the H^∞^ norm of a given closed-loop transfer function and a stochastic optimal control problem (Glover and Doyle, 1988). It has also been shown that the robust control directly connects to the risk-sensitive control via results on maximizing an entropy integral (i.e., the terminal time *N* → ∞). In addition, when the risk sensitivity parameter is equal to zero (in a risk-neutral case), the risk-sensitive control has been identified as an OFC problem. Although, in contrast to previous studies, the MMFC in this paper is derived as a time-varying controller, it is the same as OFC at two conditions: *N* → ∞ and γ → ∞. Thus, a risk-sensitive OFC seems to be a specific case of MMFC with Gaussian uncertainty. However, when there is uncertainty in the equations of motion themselves (e.g., the dynamics of a power tool such as a drill or a screwdriver are different from those of a can, resulting in strikingly different relationships between states and motor commands), structural uncertainty cannot be represented by a Gaussian distribution, and these different structures must be identified and learned (Orban and Wolpert, 2011). The MMFC proposed in this paper can handle the structural uncertainty. However, exploratory risk-taking is directly related to uncertainty in decision-making modulation (Doya, 2008), and the decision making itself may directly relate to motor control systems (Selen et al., 2012). However, the uncertainty problem may not be completely equivalent to the risk-taking problem, because the problems are distinguishable and could be identified as two independent problems (Bach et al., 2011).

### Learning process for motor adaptation

Feedback, adaptation, learning, and evolution have been identified as instances of wide sense adaptation, where sensory information is integrated and employed to change the control signals in various techniques and timescales (Karniel, 2011). Adaptive control is the change in the parameters of the control systems generated after the observation of previous control and sensory signals, and learning control is a structural change in the control system to generate a new type of behavior. In human studies, when we perform new or uncertain motor tasks, performance has been found to vary in accordance with the learning process (Shadmehr et al., 2010). Smith et al. reported that adaptation exhibited multiple timescales, driven by fast and slow processes (Smith et al., 2006). They suggested that the fast process, which decays quickly, is strongly affected by errors, but does not produce motor memory, whereas the slow process, which shows little decay, is weakly affected by errors but produces motor memory. On the other side, there are different mechanisms for adapting to stable and unstable dynamics (Osu et al., 2003). It has been proposed that adaptation learning is achieved by a combination of impedance control and an inverse dynamics model. In the early phase of learning, the impedance control also contributes to the formation of the inverse dynamics model, and helps to generate the necessary stability (Franklin et al., 2003b). Previous studies have shown that the function of the fast learning process is to increase the robustness of motor control systems, thereby improving their stability, and the internal model is obtained from multiple trials by impedance control during the slow learning process. We consider that the fast process is provided by instances of feedback and adaptation, whereas the slow process is achieved by adaptation and learning concepts. Thus, we propose MMFC as a robust control to increase the familiarity of both the uncertainty and the impedance in the adaptation of the fast process to improve the stability and reduce the error. The internal model, if it could improve the stability while achieving the task, would learn the actual dynamics across multiple trials, thereby decreasing the uncertainty in the learning of the slow process. Thus, it was recently proposed that complex behaviors in unstable dynamics cannot be explained in terms of a global optimization criterion, but rather require the ability to switch between different sub-optimal mechanisms (Zenzeri et al., 2014). We have assumed that the difference between the adaptation and learning mechanisms of stable and unstable dynamics requires that the internal model be represented in different forms, depending on the behavioral policies, off-policy and on-policy algorithms such as Q-learning and SARSA, respectively (Sutton, 1992). For example, unstable dynamics may require a deterministic behavior with an off-policy algorithm, because the cost (or reward) is assumed to be optimized through multiple trials fixing the policy to achieve the motor task in the unstable dynamics. That is, the estimated costs in any trials are required to converge to a value, similar to the idea of the worst-case design in the MMFC. In contrast, stable dynamics are assumed to require stochastic behavior with the on-policy algorithm, because it seems the best way to access the dynamics according to estimations by each trial. In addition, the off-policy algorithm has been recognized as an alternate strategy named “good-enough” control, in which the organism uses trial-and-error learning to acquire a repertoire of sensorimotor behaviors that are known to be useful, but not necessarily optimal (Loeb, 2012).

### Conflict of interest statement

The author declares that the research was conducted in the absence of any commercial or financial relationships that could be construed as a potential conflict of interest.
